# Medical Education during COVID-19: Response at one medical school 

**DOI:** 10.30476/jamp.2020.88744.1351

**Published:** 2021-07

**Authors:** SATEESH BABU ARJA, LANNY WILSON, SAMIR FATTEH, PRAVEEN KOTTATHVEETIL, AMIN FATEH, SIREESHA BALA ARJA

**Affiliations:** 1 Avalon University School of Medicine (AUSOM), Curacao

**Keywords:** COVID-19, Medical education, Teaching, Assessments, Feedback

## Abstract

**Introduction:**

The COVID-19 pandemic has caused a significant toll on healthcare across the globe. The pandemic caused many other consequences, including economic implications and teaching consequences, notably in higher education throughout the world. COVID-19 and the resulting closure of university campuses have had many impacts on Health Professions Education (HPEd), affecting all aspects, including teaching methods, assessment methods, curricula, student-teacher relationships, student selection processes, and student well-being. It has had significant effects on the setting in which students are required to learn more skills such as psychomotor skills. This manuscript aims to investigate the changes implemented in medical education during the pandemic and describe one medical school's response to medical education changes during the pandemic.

**Methods:**

This study was a combination of a case study done by in-depth investigation of the current context at one medical school during the pandemic and action research done by gathering information to change a condition in a particular place.

**Results:**

Many changes were implemented in medical education, including online teaching for basic science courses (first two years of the program) and online assessments (video-based remote proctoring) in the program's first two years. Such courses as Clinical Skills are using telemedicine/telehealth concepts in training. There were changes such as video-based remote proctoring of NBME shelf-examinations even in the assessments of clerkships/clinical rotations.

**Conclusions:**

Adaptations of medical education during this pandemic is highly dependent on technology. Most of the changes will be practiced until the campuses are open. We need to understand that these changes were made over this unprecedented period, i.e. the pandemic as an emergency rather than as a normal change process.

## Introduction

COVID-19, a disease caused by a novel coronavirus, has resulted in a pandemic that has spread to nearly every country worldwide ( 1). The COVID-19 pandemic has caused a significant toll on healthcare and healthcare workforce across the world. Countries have commenced extreme measures to prevent the disease's further spread, including highly limiting lockdowns and selective allowances for movement ( 2). The pandemic has brought about a lot of consequences, including economic and educational consequences. Higher education has been significantly affected worldwide. The world is busy tackling healthcare and economic consequences. One aspect of this pandemic that has not been explored yet is the psychological consequences. Medical education is not an exception to this bumpy ride during this difficult time. The COVID–19 pandemic has changed the way we do medical education for the near future. Both learners and medical educators, especially medical educators involved in patient care, who must handle teaching and, at the same time, patient care, have been affected. The stress and burnout of educators and learners should be given special attention. The stress on learners can be mitigated by providing the proper supporting systems to learners. If we, as educators, can identify that learners are also going through tremendous stress, we should help provide flexible solutions to students as important stakeholders ( 2).

If we reflect on ourselves in the mirror, what we have done to medical education in the past during pandemics, we can understand the current situation better. During the pandemic of Severe Acute Respiratory Syndrome (SARS), there were reports that some Chinese medical schools had formally canceled the bedside teaching and delayed the examinations ( 3). During the SARS pandemic, some medical schools in China even implemented online Problem-based Learning (PBL), which is still being utilized. Even undergraduate students were not allowed into teaching hospitals in Canada during the SARS pandemic ( 4) at the University of Toronto. However, students were allowed to attend university lectures and tutorials if they had no contact with a clinical site for ten days. The literature on Middle Eastern Respiratory Syndrome (MERS) suggested the following key points be followed: instant termination of clinical rotations, coherent decision making on a university closure, use of information technology, constant communication with hospitals, and open communication with faculty, students, and staff ( 5). There was even literature suggesting that Continuing Medical Education (CME) changed during the SARS pandemic. During major outbreaks of infectious diseases, CME providers should maintain regular contact with public health authorities and learners ( 6). Did we as a community of medical education and as medical educators learn anything from this? 

The COVID-19 pandemic has pushed the medical educators and medical schools to change their teaching methods, curricular structure, and examinations. Closure of medical schools, closure of libraries, and restrictions on access to clinical teaching facilities led to various medical schools' varied responses ( 7). COVID-19 has had a huge effect on teaching throughout the world ( 8). Even the accreditation/regulation bodies have insisted maintaining academic standards and integrity of assessments in medical schools with limited disruption to medical education. Further, the response of medical schools to the Covid-19 pandemic is unprecedented. The COVID-19 pandemic has caused disruptions to the healthcare workforce and healthcare system, and medical education worldwide ( 9, 10). The COVID-19 pandemic has necessitated a significant and rapid shift in teaching and learning strategies across all aspects of health education around the globe. Particularly, the need for physical and social distancing has severely limited conventional in-person lectures and teaching methods and encouraged a shift to online and virtual teaching modalities ( 11- 14). Virtual teaching environments present a unique set of opportunities and limitations that have been increasingly utilized over the past decade in health professional education for distance education and courses using blended learning models ( 15). 

This manuscript aims to explore the changes implemented in medical education during the pandemic and describe one medical school's response to medical education changes during the pandemic. We would like to share our experiences for the past four months at Avalon University School of Medicine (AUSOM).

## Methods

This study was a combination of a case study approach and action research. As part of the case study, an in-depth investigation was conducted across the community (all stakeholders, including teachers, preceptors/clinical faculty members, administrators, deans, and students) of AUSOM. As part of the action research, information was gathered to change a condition in a particular place at AUSOM during the pandemic. 

All changes made to medical education at AUSOM are approved by the faculty senate, curriculum committee, and quality assurance committee. AUSOM has a four-year Doctor of Medicine (M.D) program consisting of basic science courses, including clinical skills in the first two years of the program. The basic science campus is located on the beautiful Caribbean island of Curacao. Clinical rotations/clerkships are covered in the latter two-years of the program. Students do clinical rotations at teaching hospitals affiliated with Avalon across the U.S.A. in Chicago, Illinois; Beckley, West Virginia; and Phoenix, Arizona. Changes were made in delivering the medical education committed to continuing students’ learning with as little disruption as possible.

## Results and Discussion

Results have shown that the following changes were implemented in medical education during the pandemic at AUSOM. 

### Medical Education at AUSOM during the COVID-19 pandemic

#### Basic science instruction

Literature suggests that online teaching has shown to be effective and improves student learning, only if provided with the appropriate faculty development activities. Since the academic administrators know that it will be online teaching for basic science courses just before the lockdown was implemented on Curacao's island, faculty development activities were conducted to guide the faculty members on using the technologies. The Chief Information Technology (IT) officer of Avalon conducted these faculty development activities for all faculty members. The basic science or pre-clerkship instruction moved on to online teaching as campuses were closed. Some of the online platforms used by medical schools are Google classroom/Google meet, Zoom, and WebEx. We at AUSOM used the Google classroom/Google meet platform. This platform can be used if the total number of students per class is under 250, and every user needs to have a Gmail account under the G-suite domain. The other requirement is a laptop/iPad/desktop with a camera and microphone access. Preclinical instruction aims to continue with online live didactic lectures, small group discussions, and virtual labs. The preclinical program has been delivered online since March 17^th^, 2020, since the local government and public health department instructed us to close the schools and universities on March 16^th^. The didactic lectures and group discussions are conducted on the Google classroom/Google meet platform. We are using synchronized instruction. The live lectures are conducted as per the class schedules. The live lectures are provided with a ten-minute break for every fifty minutes of the lecture. Besides these live lectures, students also have access to recorded video libraries through ‘Lecturio’ platform (commercial videos). The combination of synchronous and asynchronous instruction is working out well for our students. Small group discussions and PBL sessions were conducted using google sheets and google documents. Each group deposits the information or work done from all partners of that particular group and is supplemented with discussions.

Virtual labs are conducted using various resources. For example, anatomy uses resources like Grant’s Dissector Videos in addition to resources like Osmosis and Complete Anatomy ( 16 , 17 ). Teaching clinical skills is continued during the lockdown and after the lockdown. During the lockdown, teaching clinical skills was continued by using various videos, including Jove Science Education ( 18 ). Other resources used include the telemedicine/telehealth concept and the communication skills set developed by the Academy of Communication in Healthcare (ACH) ( 19 ). Once lockdown was over, we used video-based teaching with Standardized Patients (SP) in the clinical skills course. We used virtual microscopy for histology and pathology. For histology, shotgun videos available on YouTube are being used. For pathology, medical school digital pathology is being used ( 20). 

#### Assessments in basic science courses

Lockdown and travel restriction due to COVID-19 prevented in-person examinations. Our assessments in basic science courses have continued to be online assessments. Students are taking the formative and summative assessments on Examsoft software. As students are taking these assessments online remotely, we have taken the utmost precautions to maintain assessments' integrity. All evaluations are timed. All online assessments are proctored using video-based proctoring. Students must have two gadgets; one device (either a laptop, desktop, or iPad) for doing the exam. Examsoft has a specific feature where students cannot open any other program/software while they are on the exam. Another device (either smartphone or iPad) is required for video-based monitoring through Google classroom/Google meet. Each proctor monitors approximately ten to fifteen students. All students are required to sign the consent to monitor them remotely using the Google meet platform. Lab examinations are being conducted online using the Objective Structured Practical Examinations (OSPE) and SP-based assessments in the clinical skills course using video calls. In clinical skills, online assessments are conducted by SP-based assessments on the Google classroom/Google meet platform.

### Support to the learners

#### Pandemic preparedness

The emergency management committee conducted a meeting immediately after hearing the Coronavirus situation. They drafted the policy and regulations, then communicated with faculty and students regularly. In regards to traveling, we recommended that students follow the guidelines of the Centers for Disease Control and Prevention (CDC), U.S.A., and the Public Health Department of Curacao, which can be accessed on their websites.

Once the local public health department closed schools and universities, we moved on to online instruction, and further guidelines were given to faculty and students. Once the lockdown was implemented on the island, the school also helped students and faculty with shopping. The school managed to arrange grocery store trips for the students and faculty who were on the island. In order to maintain physical distance and hygiene, the following precautions were taken; 6 students per trip; signs and information in the bus; sanitizer, gloves, and towels; and the driver had his manual with guidelines and instructions to follow. 

#### Feedback to the learners

Feedback to the learners is provided regularly. Students can schedule appointments with basic science faculty members, and they can meet on the Google meet platform at the scheduled time. Feedback is provided to the learners immediately after each assessment. Students can see the rationale for each question incorrect on Examsoft after completing their assessments since they are provided with a review password. Feedback provided is effective if the feedback is specific and provided within a given time frame ( 21 ). In addition to the feedback provided through Examsoft software, students can request appointments with the faculty members to get academic advice on the Google meet platform as described above. Each department also mandated that faculty members provide academic guidance and support at scheduled intervals on the Google meet platform with each student. Each faculty member is assigned to ten to fifteen students to provide academic advice and counseling as needed. 

#### Peer-tutoring

At AUSOM, peer-tutoring has been continued even during the pandemic. Students interested in tutoring contact the Student Government Association (SGA), which will inform the associate dean of basic science. Senior tutoring students are selected by faculty and the associate dean of basic science, based on academic criteria and tutoring interest. Students participating in tutoring receive monetary compensation from the school. During the pandemic, we continued the peer-tutoring sessions. The only difference is that students are scheduling the meetings with their tutors online using Google meet platform rather than in the classroom. 

### National Board of Medical Examiners (NBME)

#### comprehensive basic science examination

AUSOM requires all students to take NBME Comprehensive Basic Science Examination (CBSE) and pass with a 197 or higher score before moving on with clinical rotations. The same has continued with our students during the pandemic. Since Prometric test centers are closed, we had to take steps to deliver the CBSE exams with an alternative test strategy as guided by NBME. CBSE examinations are conducted with remote proctoring on Zoom. Students will need two devices; one to run the exam (computer/laptop) and one to run the Zoom application (phone/tablet). Students should ensure that no recording programs are running on their device before doing workstation certification for NBME and before beginning the test day. Students can communicate with the proctor during the test using chat before launching the exam and completing the exam. During the examination, students will need to use their cell phones to text or call the proctor. Each proctor is allowed to monitor only ten students at a time. Students need to download the Zoom application before the test day and enable video when they join Zoom. All students taking these exams are notified in advance with the required instructions. IT support is offered when needed by the students. 

#### Clinical Rotations

Our students do rotations at affiliated hospitals in Chicago, Illinois; Beckley, West Virginia; and Phoenix, Arizona. The core rotations have continued with minimal disruptions at Chicago and West Virginia. The hospitals at Phoenix, Arizona, stopped rotations during the pandemic for medical students for the time being. But fortunately, we had a very minimal number of students in Phoenix, Arizona. When students have restrictions on entry to hospitals and direct involvement in patient care, physicians engage these students in outpatient settings in their clinics. The clerkships adapted to cover the educational objectives, core clinical encounters, content, and assessments, and they were delivered. If students have been enrolled in clinical rotations, we want every one of them to complete the rotation with their assigned preceptor. Circumstances may have changed, but the preceptor-student mentorship has not. Students are required to complete the rotation with preceptors in their clinics. 

In addition to students attending clinics, students were also involved in telemedicine with their preceptors. Online learning resources complemented this outpatient/clinic teaching. Students must do at least 3 hours/day of video-based library resource ‘Lecturio’ (5 days/week), and we expect a student to get a certain grade before going to the next lesson on ‘Lecturio’ for recall questions after each video (formative assessments). Students are also required to write reflective writing at the end of each week regarding their activities. The clinical department monitor students’ engagement in the video-based library. 

#### Students’ assessments during clinical rotations

NBME shelf-examinations have continued for core rotations (20% of the grade). Since Prometric test centers are closed, shelf-examinations are conducted using remote proctoring by video call using the Zoom application. 

Physician evaluation of students for core rotations (80% of the grade) has continued since students are under the physicians’ observation. Also, students’ engagement in the video library and weekly reflective writings play a role in evaluation. 

Physician evaluation of students for elective rotations (100% of the grade) has continued since students are under the physicians’ observation. Also, students’ engagement in the video library and weekly reflective writings play a role in their evaluation.

#### Objective Structured Clinical; Examinations (OSCE)

Passing the OSCE exam is a requirement for all Avalon students to graduate. Students are allowed to take the OSCE exam only after completing core rotations. Due to COVID-19 and travel restrictions, physical OSCE was suspended for the time being. Virtual OSCE and OSCE by Zoom replaced the original OSCE. Because we cannot conduct the face-to-face exam format, the student will be conducting a telemedicine interview with the standardized patient. Then students type out the encounter after each telemedicine interview. For the virtual exam, students will have to verbalize to the SPs the maneuvers that they would do if students were in a face-to-face encounter. The SPs will listen, and after students have finished describing the maneuver, they will report to the student if the findings are normal or any other findings. Students are provided with all guidelines and trained on how to verbalize the physical exam. Students will be asked to type out Subjective, Objective, Assessment, and Plan (SOAP) notes during the Zoom conference in a timed format. 

#### Selection of students into medical school/ admissions

Literature suggests that Mini Multiple-Interviews (MMIs) are more reliable and valid. Many articles are in support of MMIs, and MMIs have proven validity and reliability ( 22 ). But many studies found no additional benefit or advantage of conducting MMIs. Some papers found no difference in students' acceptance rates either through traditional interviews or MMIs ( 23 , 24 ). In an interesting study with a hybrid interview model combining traditional and multisampling formats, favorable results were found ( 25 ). 

Some of the stations that can be used in the MMI process include, but are not limited to, experiences outside the school, how a student is thinking about international medical graduate work in rural areas, a student withdrawing from the university’s course, and how he/she can counsel the student, selecting a dean for the medical school and so on. There is no literature suggesting MMIs using Skype interviews. AUSOM implemented MMIs through Skype in 2017. AUSOM is located on the Caribbean island of Curacao. Students are mostly international students. Therefore, face-to-face interviews are not possible. 

Students are assessed during the interview process by skype interview in diverse areas, including motivation and preparation, ethical decision making, knowledge of health care systems, social and cultural competence, and critical thinking. The other areas that are included in the interview process are compassion, empathy, maturity, teamwork, and commitment to the community. Then the interviewers rate and assess the students based on students’ responses. At AUSOM, an interview form was created in 2017 with various questions depicting MMIs' different scenarios. Due to the current COVID-19 crisis, medical schools globally face the possibility of not running face-to-face MMIs. 

## Conclusions

All changes made to medical education during this pandemic might not be permanent. Some of the changes will be in practice until the campuses are open. Some of the changes might be permanent. These changes leaning on online instruction and online assessments should be considered as changes made during the crisis rather than changing to online teaching as a standard change process under normal conditions. Online instruction may not be a permanent solution for medical education and medical schools. In the future, medical schools should consider a combination of online instruction, face-to-face modalities, group discussions, and skill development labs. Faculty development activities play a key role when moving on with any instructional methods using technology. Motivating and encouraging students are both critical components of success during these trying times, and communication is the key.

This study's main limitation is that this study was aimed as a case study at one of the medical schools in the Caribbean. The conclusions might not be generalizable. But the good practice points mentioned in this manuscript could be implemented at other institutions. The introduction captures the history of other pandemic situations like SARS and MERS and how the society as a whole and especially the medical community managed it. The manuscript narrates how the medical school's teaching evolved to meet the challenge. In the discussion, we continued to describe many aspects of our teaching, from basic sciences to clinical sciences. In general, much of it is what all the medical schools adopted in the pandemic crisis. But this is a brief report written recounting an experience of one medical school during the COVID-19 pandemic.
